# High prevalence of *Bordetella pertussis* in children under 5 years old hospitalized with acute respiratory infections in Lima, Peru

**DOI:** 10.1186/s12879-015-1287-z

**Published:** 2015-12-02

**Authors:** Ivana Pavic-Espinoza, Sandy Bendezú-Medina, Angella Herrera-Alzamora, Pablo Weilg, María J. Pons, Miguel Angel Aguilar-Luis, Verónica Petrozzi-Helasvuo, Juana del Valle Mendoza

**Affiliations:** Centro de Investigación de la Facultad de Ciencias de la Salud. Universidad Peruana de Ciencias Aplicadas - UPC, Av. San Marcos cdra. 2. Cedros de Villa, Lima, Peru

**Keywords:** Bronchiolitis, *Bordetella pertussis*, Infants, Vaccine

## Abstract

**Background:**

Pertussis diagnosis may go unrecognized when other pathogens, such as respiratory syncytial virus (RSV) circulate.

**Methods:**

A prospective cross-sectional study was conducted in Lima, Peru from January 2009 to September 2010. A total of 596 children under 5 years old admitted with clinical diagnoses of acute respiratory infections were test for *B. pertussis* and RSV detection by polymerase chain reaction (PCR).

**Results:**

The pertussis toxin and IS481 genes were detected in 19.12 % (114/596) of the cases and the respiratory syncytial viruses (RSV-A and RSV-B) were identified in 17.28 % (103/596) of patients. Infants under 3 months old were the most frequently affected by this pathogens in 43 % (49/114) and 35.9 % (37/103) respectively. An increase of *B. pertussis* was observed from February to March and from October to November with a Seasonal index between 1.32 and 1.51 and 1.24–3.5 respectively.

**Conclusions:**

Epidemiologic surveillance for *B. pertussis* is essential in Peru, especially in children that could most benefit from the vaccine. *B. pertussis* should be suspected in infants hospitalized for acute respiratory symptoms for early treatment and prevent complications.

## Background

Pertussis is an endemic vaccine-preventable disease with the highest morbidity and mortality in the youngest infants [1]. Worldwide, there are an estimated of 16 million cases of pertussis, 95 % of which occur in developing countries, resulting in about 195 000 children deaths per year [1, 2]. In the last years, an increase in reported cases of pertussis has been noted, even in countries with high vaccination coverage [3–6].

*Bordetella pertussis* is a fastidious gram-negative coccobacillus which causes Pertussis disease, a highly contagious infection of the human respiratory tract also known as “whooping cough” [3, 5]. Pertussis is characterized by three phases: catarrhal, paroxysmal, and convalescent; being the most infectious periods the catarrhal and early paroxysmal phases [7]. This classic presentation is well-known, but is observed less often since the start of immunization [4].

The standard diagnostic criteria for *B. pertussis* identification and epidemiological surveillance are culture and molecular techniques such as polymerase chain reaction (PCR). The DNA amplification techniques (e.g., PCR) for *B. pertussis* detection are faster, and have increase the sensitivity by approximately 19 % the overall percentage of laboratory-confirmed cases, being the preferred method [8, 9]. However, in clinical practice the diagnosis is generally reached without microbiological confirmation leading to a possible lack of clinical awareness to start early treatment and prevent complications [10].

Pertussis can be especially difficult to diagnose in children under 1 year of age during winter season, when other pathogens, such as respiratory syncytial virus (RSV) or Influenza, are prevalent. In these difficult cases, pertussis acute respiratory symptoms can overlap with those of bronchiolitis or other unspecific acute respiratory infections [10, 11]. This is especially worrisome in infants too young to be immunized in whom atypical and more severe presentations have been reported, often requiring hospitalization for respiratory or other complications [7, 12, 13].

In Peru, pertussis is a major health problem that has been raising in the last 5 years [14]. Furthermore, the most affected are infants under 1 year old representing 38 % of cases, despite a national immunization coverage of 92 % in this age group [15, 16]. Currently, the “whole cell” *B. pertussis* vaccine (DTwP) is the only available presentation in Peru; and the national coverage level for this vaccination is 88.3 % for the 3 doses of the pentavalent vaccine (DTwP-Hib-HepB) according to the 2014 epidemiology reports [17].

To study the Pertussis epidemiology in Peru is essential in order to understand the real impact of the disease, especially in the most vulnerable population. The aim of this study is to determine the prevalence, epidemiological and clinical characteristics of *B. pertussis* and Respiratory syncytial virus cases in infants under 5 years old hospitalized with acute respiratory infections in a Peruvian hospital between 2009 and 2010.

## Methods

### Patients

A prospective cross-sectional study was conducted in children under 5 years old admitted to *“Hospital Nacional Cayetano Heredia. Lima - Peru”* with diagnosis of acute respiratory infection (ARI). A total of 596 patients were studied from January 2009 to September 2010. The study region had a representative population, since Lima is recognized as *B. pertussis* endemic area and has a vaccine coverage similar to the national reports.

Epidemiological and clinical features were registered, including: age, gender and clinical symptoms (fever, rhinorrhea, cough, respiratory distress, malaise, wheezing, pharyngeal congestion, expectoration, vomiting, diarrhea, among others).

This study was approved by the Research Ethics Board of the “*Hospital Nacional Cayetano Heredia and Universidad Peruana de Ciencias Aplicadas”*. Informed written consent was given by the parents or legal guardians of the children before enrolment.

### Samples

Two nasopharyngeal samples were obtained per patient. The first one, by inserting a swab into both nostrils parallel to the palate (calcium alginate swab, USA) and a second swab for the posterior pharyngeal and tonsillar areas (Viral Culture, Becton-Dickinson Microbiology Systems, MD, USA). Both nasal and pharyngeal swabs were placed into the same tube containing viral transport medium (a minimal essential medium buffered with NaHCO_3_ and supplemented with 2 % fetal bovine serum, penicillin and streptomycin 100 U/ml, amphotericin B 20 μg/ml, neomycin 40 μg/ml). The samples were then stored at 4 °C until being sent to the Laboratory of molecular biology at “*Universidad Peruana de Ciencias Aplicadas (UPC)*”. On receipt of the samples the swabs were discarded and tubes were centrifuged to pellet the cells, which were re-suspended in 0.8 ml of PBS 1X. Two aliquots of 200 μl of each fresh specimen were used for the extraction of nucleic acids and 200 μl for bacterial culture.

### DNA extraction

DNA was extracted from a volume of 200 μl of each samples using a commercial kit (High Pure Template Preparation Kit, Roche Applied Science, Germany) according to the manufacturer´s instructions. DNA extraction was assayed immediately or stored at −80 °C until use.

### PCR amplification

The presence of *B. pertussis* was determined using two PCR assays, each specific for an independent region of the *B. pertussis* genome. A fragment of 191-bp of the pertussis toxin S1 gene (PTxA) was amplified using the primers PTp1: 5´-CCAACGCGCATGCGTGCAGAxTTCGTC-3´ and PTp2:5´-CCCTCTGCGTTTTGATGGTGCCTATTTTA- 3´ [18]. Meanwhile a 145 bp fragment of the insertion sequence IS481 was amplified using the primers IS481F: 5'-GATTCAATAGGTTGTATGCATGGTT-3' and IS48R: 5'-TTCAGGCAGACAAACTTGATGGGCG-3´ [19]. The described procedures were slightly modified as follows: Fifty μl of reaction mixture containing 25 ul ready mix enzyme (Taq polimerase, 2.5 mM Mg Cl2; 15 mM Tris/HCl PH 8.3, 50 mM KCl, 200 uM each deoxynucleotide) (Kappa Biosyste), 20 pmol each primer (Macrogen-Korea), water and 5 ul DNA were amplified using a pre-denaturation of 5 min at 95 °C, followed by 55 cycles of denaturation for 1 min at 95 °C, annealing for 1 min at 55 °C and elongation for 45 s at 72 °C, with a final elongation of 10 min at 72 °C. The presence and size of amplification products were analysed by electrophoresis on 2.5 % gel agarose, containing 3 μg/mL of ethidium bromide, and photographed under ultraviolet illumination. The amplified products were sequenced (Macrogen, Seoul, Korea).

Respiratory syncytial virus (RSV-A and RSV-B) were identified by multiplex RT-PCR as previously described by Coiras et. al., [20].

### Statistical analysis

Qualitative variables were reported as frequencies and percentages. All analyses were processed with the IBM Statistical Package for the Social Sciences (SPSS) software version 21.0 (SPSS, Chicago, IL, USA). The chi-square test (χ^2^-) was used to assess associations between categorical variables while z-Test was used to 30 is significant. A *p*-value <0.05 was considered statistically significant. A seasonal index was calculated for *Bordetella* and Respiratory syncytial virus PCR-confirmed cases from January 2009 to September 2010. Seasonal indexes were calculated dividing the monthly frequency of confirmed cases by the average of cases per year.

## Results

A total of 596 children under 5 years diagnosed with an acute respiratory infection were admitted to the *“Hospital Nacional Cayetano Heredia. Lima - Peru”* from January 2009 to September 2010. The pertussis toxin and IS481 genes were detected in 19.12 % (114/596) of the cases. Respiratory syncytial viruses (RSV-A and RSV-B) were identified in 17.28 % (103/596) of patients. Co-infections between *B. pertussis* and RSV-A were observed in 14 patients and only one sample was positive for *B. pertussis* and RSV-B (Table 1).

**Table 1:** General characteristics of *Bordetella pertussis* and Respiratory Syncytial Virus cases

Characteristic	Total ARI^a^ patients	*Bordetella pertussis*	RSV^b^
	Frequency (*n* = 596)	Frequency (*n* = 114)	Frequency (*n* = 103)
	N (%)	N (%)	N (%)
Gender			
Female	243 (40.8)	52 (45.6)	46 (44.7)
Male	353 (59.2)	62 (54.4)	57 (55.3)
Age			
Newborn (≤28 days)	112 (18.8)	17 (14.9)	11 (10.7)
29 days – ≤ 3 months	121(20.3)	32 (28.1)	26 (25.2)
3 – 5 months	82 (13.8)	13 (11.4)	11(10.7)
6 – 11 months	115(19.3)	20 (17.6)	26 (25.2)
1 – 5 years	166 (27.9)	32 (28.1)	29 (28.2)
Contact with another people with ARI^a^			
Yes	353 (59.2)	67 (58.8)	59 (57.3)
Not	243 (40.8)	47 (41.2)	44 (42.7)

*ARI* ^*a*^ acute respiratory infection

*RSV* ^*b*^ respiratory syncytial virus

Positive samples for *B. pertussis* and RSV were analyzed according to age distribution, and infants under 3 months old were the most frequently affected in 43 % (49/114) and 35.9 % (37/103) respectively. A similar sex distribution was observed in both groups. Moreover, around 59 % of enrolled children had a previous contact with another patient with acute respiratory infections. An equivalent proportion of household contacts was observed for *Bordetella pertussis* and RSV positive samples (Table 1).

A similar clinical symptoms frequency was observed between patients with *B. pertussis* and RSV. The most common symptoms in both groups were fever, cough, rhinorrhea and respiratory distress, all of them present in more than 60 % of cases. However, among the patients with a positive RSV sample a higher rate of Rhinorrhea 88.35 %, Respiratory distress 76.70 % and pharyngeal congestion 33.98 % was observed, in comparison with the Pertussis-positive group (Table 2).

**Table 2:** Clinical symptoms observed in patients with positive *B. pertussis* and RSV by PCR

Clinical symptoms	Total of patients	Patients positive for *Bordetella pertussis*	Patients positive for RSV
	Frequency (*n* = 596)	Frequency (*n* = 114)	Frecuency (*n* = 103)
	N (%)	N (%)	N (%)
Fever	596 (100)	114 (100)	103 (100)
Cough	448 (75.2)	82 (71.9)^a^	92 (89.32)*
Rinorrhea	448 (75.2)	90 (78.9)	91(88.35)
Respiratory distress	366 (61.4)	69 (60.5)^a^	79 (76.70)*
Wheezing respiratory	230 (38.6)	40 (35.1)^a^	59 (57.28)*
Malaise	150 (25.2)	28 (24.6)	24 (23.30)
Pharyngeal congestion	150 (25.2)	25 (21.9)^a^	35 (33.98)*
Expectoration	142 (23.8)	28 (24.6)	30 (29.13)
Vomits	79 (13.3)	16 (14)	16(15.53)
Diarrhea	71(11.9)	13 (11.4)	15 (14.56)
Asthenia	52 (8.7)	13 (11.4)	9 (8.74)
Conjunctival congestion	23(3.9)	5 (4.4)	5 (4.85)
Abdominal pain	21(3.5)	2 (1.7)	2 (1.94)
Headache	16 (2.7)	3(2.63)	4 (3.88)
Otalgia	6 (1.0)	2 (1.75)	1 (0.97)
Myalgia	6 (1.0)	1(1.75)	1 (0.97)

* z-Test: Patients positive for *Bordetella pertussis* vs Patients positive for RSV, *p* < 0.05

Others (<10 % of cases): Ear pain, photophobia, conjunctival congestion, abdominal pain

lymphadenopathy, fatigue, headache, myalgia, skin rash

^a^3 children died, one of them in the *B.pertussis* infection group

Pneumonia was the most frequent clinical diagnosis in 32.38 % (193/596) of the total of patients hospitalized with acute respiratory infections. The diagnosis of Bronchiolitis was more common in children with a positive sample for RSV in 20.39 % (21/103). On the contrary, the diagnosis of rhinopharyingitis 6.14 % (7/114) was more common in patients with positive *B. pertussis* (Table 3).

**Table 3:** Clinical diagnosis observed in patients with positive *B. pertussis* and RSV by PCR

Clinical diagnosis	Total of patients		Patients positive for *Bordetella pertussis*			Patients positive for *RSV*		
	Frequency	Prevalence	Frequency	Prevalence	*p*-value**	Frequency	Prevalence	*p*-value**
	(*n* = 596)	(%)	(*n* = 114)	(%)		(*n* = 103)	(%)	
Pneumonia	193	32.38	30	26.32*	0.124	44	42.72*	0.014
Pharyngitis	6	1.01	0	0	0.231	1	0.97	0.968
Rhinopharyngitis	33	5.54	7	6.14	0.754	3	2.91	0.200
Bronchiolitis	57	9.56	9	7.9*	0.327	21	20.39*	<0.05
Influenza A Infection	51	8.56	10	8.77	0.927	6	5.83	0.276
Whooping cough-like syndrome	10	1.68	3	2.63	0.378	2	1.94	0.819
Obstruction syndrome to bronchiolar	41	6.88	9	7.89	0.634	11	10.68	0.094

* z-Test: Patients positive for *Bordetella pertussis* vs Patients positive for RSV, *p* < 0.05

** χ^2^-Test

Others (1 % of cases): Sinusitis, respiratory distress syndrome, sepsis late atypical febrile seizure status epilepticus, atypical febrile seizure, gastroenteritis

**Fig. 1:**
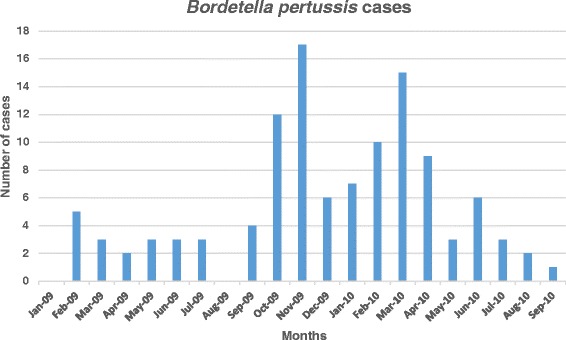
*Bordetella pertussis* confirmed cases (2009–2010)

**Fig. 2:**
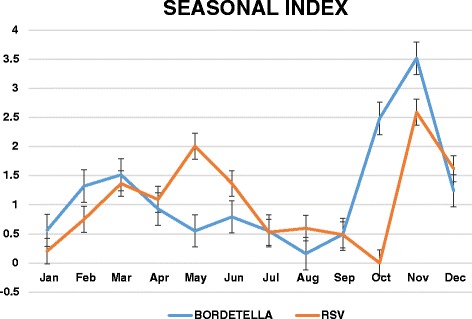
Bordetella pertussis and RSV seasonal index (2009–2010)

A higher prevalence of *B. pertussis* cases were registered between October and November 2009 and February to April 2010. (Figure 1) Seasonal indexes were calculated for *B. pertussis* and RSV positive samples separately. An increase of pcases was observed from February to March and from October to November with a Seasonal index between 1.32 and 1.51 and 1.24–3.5 respectively. A similar predominance was observed in RSV cases from November to December. However, RSV showed to be also frequent from April to June with a seasonal index between 1.09 and 2.00 (Fig. 2).

## Discussion

*Bordetella pertussis* is a strict human pathogen which causes whooping cough, an endemic illness responsible of significant morbidity and mortality, especially in infants under 6 months old [1, 2, 5]. Although regional differences exist, Pertussis represents a considerable global disease burden that has been increasing, even in countries with high vaccination coverage [2, 5, 13]. In Peru, an alarming increase of cases has been observed in the last 5 years, and 56 % of cases are reported in infants under 1 year old [12, 14–16]. This have raise especial concern since infants under 6 months old are more vulnerable to disease related complications and carry a higher mortality [7, 21, 22].

The most common clinical manifestations of *B. pertussis* infections are prolonged and paroxysmal coughing, accompanied by inspiratory stridor [1, 3]. However, several factors are known to affect the disease presentation and Pertussis diagnosis may go unrecognized when other pathogens, such as respiratory syncytial virus (RSV) or Influenza virus circulate [10, 23, 24]. A retrospective study in Italy, from a group of infants hospitalized from October 2008 to April 2010 for acute respiratory symptoms reported that most of Pertussis cases were infants under 6 months with median of 71.5 days old and a male: female ratio of 6:13 [10]. In our study pertussis toxin and IS481 genes were detected in 19.12 % (114/596) of the patients admitted with an acute respiratory infection and infants under 3 months old were the most frequently affected in 43 % (49/114) with a similar sex distribution.

Co-infection between *Bordetella pertussis* and RSV has been previously described to cause severe infections [10, 11]. A study conducted in a group of infants hospitalized for RSV bronchiolitis showed that almost 2 % of patients were co-infected with *B. pertussis* [25, 26]. In our series, co-infections were observed in 14 patients between *B. pertussis* and RSV-A and 1 sample was positive for *B. pertussis* and RSV-B. Moreover, 6 out of 9 cases of co-infections were clinically diagnosed as Bronquiolitis and *B. pertussis* was not suspected at the time of admission. Influenza virus and *B. pertussis* co-infections have been also identified as a possible pathogen present in children with community-acquired pneumonia; and the pertussis toxin-mediated suppression have been postulate to be responsible to produce more sever presentations [27, 28].

Multiple studies have reported Paroxysmal cough (76.5–91.1 %), cyanosis (46.7–81.7 %) and respiratory distress (47.8–55.7 %) as the most common symptoms in children [13, 29, 30]. However, several clinical features might help to suspect the diagnosis of pertussis in infants hospitalized for acute respiratory symptoms. [10]

One study in 2013, compared infants with Pertussis and confirmed RSV bronchiolitis; and the clinical characteristics showed that the percentage of infants with paroxysmal cough was significantly higher in infants with *B. pertussis*. Additionally, cough at admission lasted longer in infants with pertussis than in control infants. Also, fever was significantly lower in infants with pertussis, and more common in patients with bronchiolitis. In our study population, a similar clinical symptoms frequency was observed between patients with B. pertussis and RSV. The most frequently reported symptoms were fever, cough, rhinorrhea and respiratory distress, in more than 60 % of cases. However, the presence of rhinorrhea 88.35 %, respiratory distress 76.70 % and pharyngeal congestion 33.98 % was more common among patients with RSV. This higher frequency of symptoms in our study may be related to fact that more than 52 % of our patients were hospitalized infants under 6 months old.

The clinical diagnosis of Pertussis in infants can be challenging, especially in children with incomplete immunizations, and some patients may be catalogued as acute viral respiratory infections, before laboratory confirmation. Thus delaying the appropriate antibiotic treatment and isolation measures [11, 24]. In our series, pneumonia was clearly the most frequent diagnosis in 26.32 % (30/114) of the patients with positive *B. Pertussis*. However, other diagnosis were considered in this group, such as rhinopharingitis, bronchiolitis and influenza infections. In contrast, the diagnosis of Bronchiolitis was more common in 20.37 % (21/103) of children with a positive sample for RSV.

For *Bordetella pertussis* seasonality, a pattern corresponding to the summer and spring months have been reported in the southern hemisphere [13]. Comparably, a previous study in infants under 6 month of age from 2003 to 2008 in Lima, registered more hospitalizations due to whooping cough during the months of February and September. In our study, a similar distribution was observed with an increase of *B. pertussis* cases from February to March and from October to November and a Seasonal index between 1.32 and 1.51 and 1.24–3.5 respectively.

Pertussis represents a considerable disease burden in Peru and the diagnosis is complicated by the limitations of currently available diagnostic tests. Therefore, the only diagnostic tests that are recommended for case confirmation in national reporting are culture and polymerase chain reaction (PCR) [7, 31]. However, in Peru the use of PCR for surveillance was started recently in 2012 and there is still evidence of a deficient report and registration of cases that limit the analysis of the real disease burden.

## Conclusions

As in other Latin American countries, epidemiologic surveillance for *B. pertussis* is essential in Peru, especially in children that could most benefit from the vaccine. This study demonstrates a considerable incidence of *B. pertussis* in children previously diagnosed as acute respiratory infections and highlights the importance of possible co-infections that may difficult the diagnosis and prognosis of patients. There is an increasing need for further investigations to better establish the impact of the disease and improve vaccination programs especially in hospitalized children were more severe presentations have been reported.
